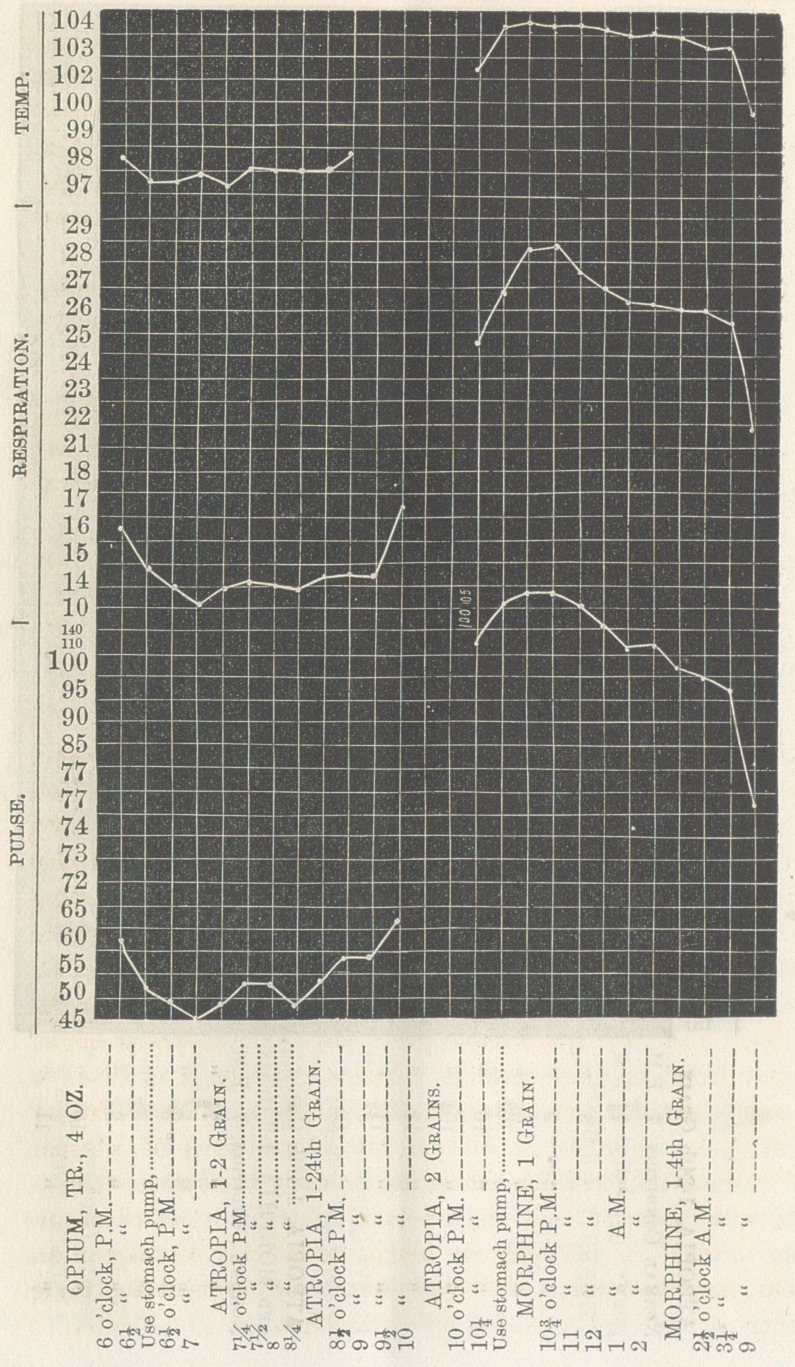# Antagonisms between Opium and Belladona

**Published:** 1871-05

**Authors:** Lyman Ware

**Affiliations:** Chicago


### ANTAGONISMS BETWEEN OPIUM AND BELLADONNA.

BY LYMAN WARE, M.D., Chicago.

Owing to the extreme contradictory opinions of eminent medical authorities, since the seventeenth century, respecting the antagonistic properties of opium and belladonna, the writer long desired to perform a few careful and exact experiments, to determine, in his own mind, to what extent, if any, the antagonisms do exist. The experiments were wholly unprejudiced, as the believers and disbelievers in the mutual antidotal action of opium and belladonna are equally esteemed for their profound and exact knowledge and teachings. If these experiments are in degree as interesting to others as to the performer, the labor of writing out the data will be fully compensated.

The experiments were performed with the sulphates of morphia and atropia, using a *Charriere* hypodermic syringe, the needle being adjusted and the piston worked by a screw, securing perfect exactness. Most of the experiments were performed upon the person of the writer, being assisted while under the influence of the larger doses by an intelligent gentleman in taking pulse respiration and temperature, in order that no mistake might possibly occur.

About fifty hypodermic injections were carefully noted, with not a single untoward symptom. There was momentary pain at the point of injection, but no special soreness subsequently.

Great care was taken: 1st. That the soulution be perfectly neutral. 2d. That no air be in the syringe. 3d. That the point of injection be loose tissue, so as to be grasped between the thumb and index-finger. 4th. That the injection be given slowly, and the finger applied for a moment at the point from which the needle is taken, to make sure that none of the solution escapes.

The observations are not all given, there being, of necessity, repetition, as many of these were repeated to confirm and fully establish the constancy with which the several phenomena made their appearance.

Prior to entering upon these experiments the writer had scarcely ever taken either of the drugs of which he made use.

Sufficient time was always allowed to intervene between the observations, in order that the system might fully recover its normal condition.

That the relationship existing between opium and belladonna may the easier be comprehended, the phenomena accompanying the action of each will be considered.

Experiments were also made with hyoscyamus and stramonium, using their alkaloids, hyoscyamia and daturia, but as their relationship to opium, in their antidotal effects, is so nearly allied to that of belladonna, the data have not been written out.

Observations after giving $\frac{1}{8}$th of a grain of morphia:

TEMP.

RESPIRATION.

In the smaller doses of atropia there is noticed a diminution of the pulsation, and in the smaller doses of morphia an increase of the pulsations. When atropia is combined with morphia, for its soporific effect, the smaller dose, $\frac{1}{96}$th or $\frac{1}{120}$th of a grain of atropia should be used with $\frac{1}{3}$d of a grain of morphia, thus obtaining the sedative effect of each, with the unpleasant effect of neither.

In comparing the physiological effects of the two alkaloids, it is at once seen that neither have a common phenomena, as in:

| MORPHIA. | ATROPIA. |
|---|---|
| The face becomes blanched; external temperature diminished; pupil contracted; dilation of capillaries; diminished respiration; pulsation diminished in frequency, increased in force; constipation of bowels; nausea; depression of vital force. | The face becomes flushed; external temperature increased; pupil dilated; contraction of capillaries; increased respiration; pulsation increased in frequency, diminished in force; relaxation of the bowels; exaltation of vital force. |

The two cases of poisoning that came under observation, where, in one four ounces of laudanum were taken, and in the other two grains of morphine, made good their recovery; although in the former case fatal syncopy occurred as the patient undertook to arise from his bed the following day. Where toxic symptoms are to be contended with, it is impossible to determine definitely how much of the antidote should be used, not knowing the amount of poison that may have been already absorbed, and without great care toxic smyptoms might result from the very antidote. Yet this danger may be in a measure avoided, by approximation of the antidote, recalling that $\frac{1}{48}$th of a grain of atropia will neutralize $\frac{1}{2}$ of a grain of morphia. The influence of either upon the system follows the administration so closely, beginning to show its effects in two minutes, in reaching its height so quickly, usually in an hour, they need only to be closely watched to prevent any toxic influence and obtain the full antidotal effect.

## Figures and Tables

**Figure f1:**
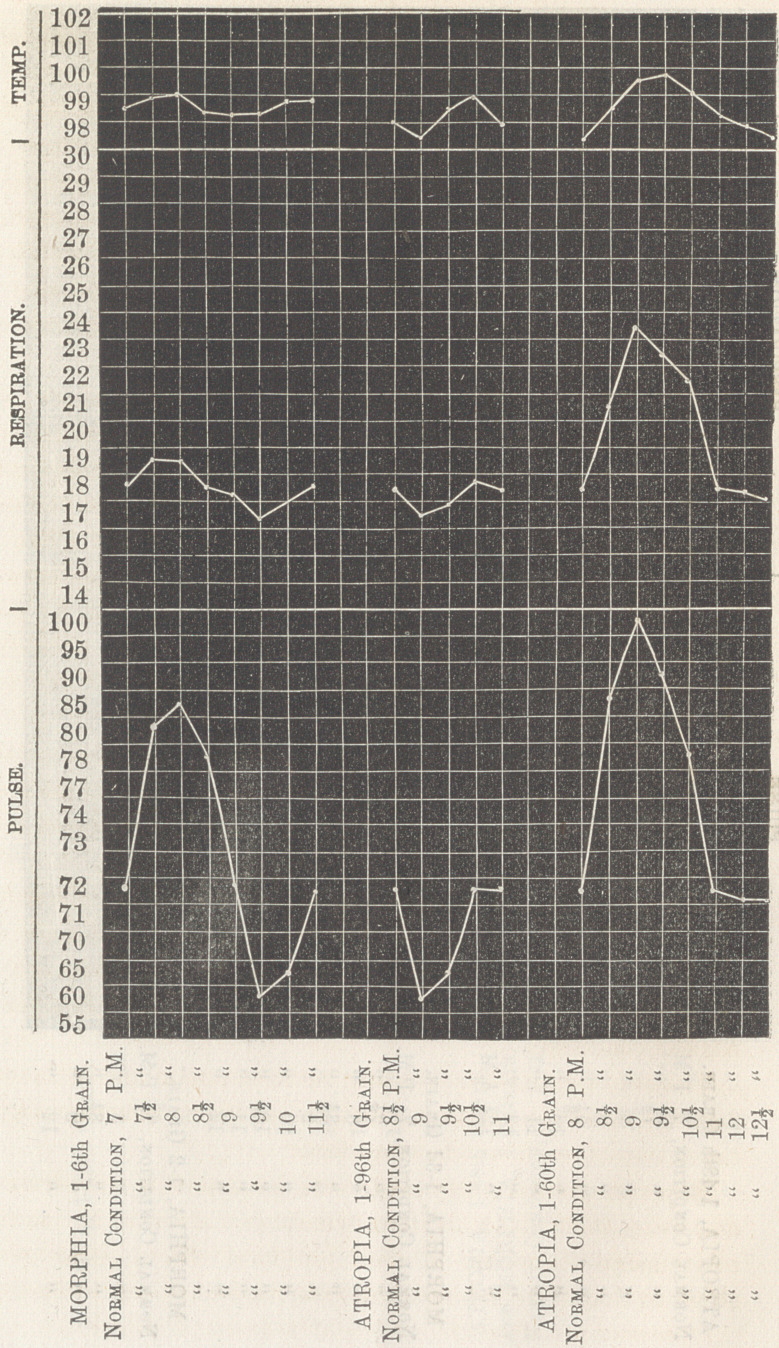


**Figure f2:**
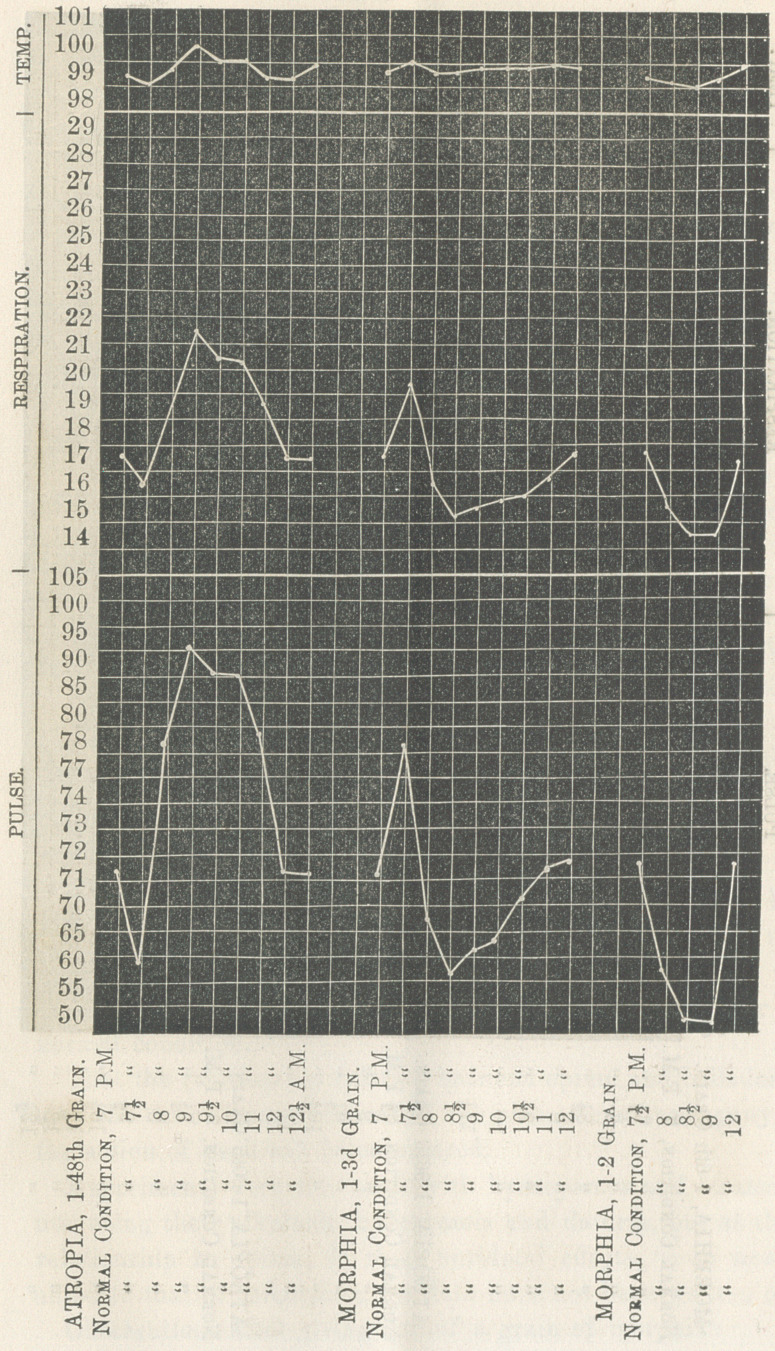


**Figure f3:**
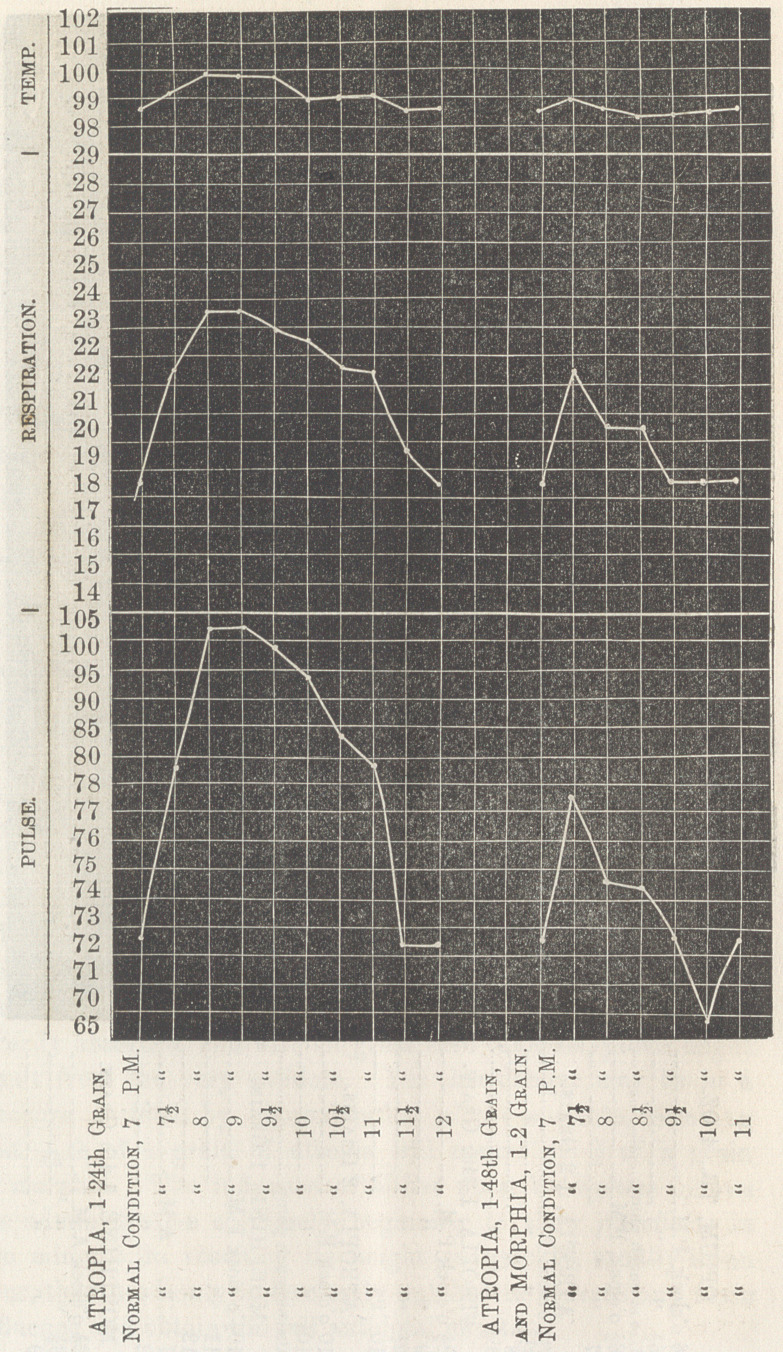


**Figure f4:**